# Heme biosensor-guided in vivo pathway optimization and directed evolution for efficient biosynthesis of heme

**DOI:** 10.1186/s13068-023-02285-4

**Published:** 2023-03-01

**Authors:** Jian Zhang, Qingbin Li, Qi Wang, Jingyu Zhao, Yuan Zhu, Tianyuan Su, Qingsheng Qi, Qian Wang

**Affiliations:** 1 National Glycoengineering Research Center, State Key Laboratory of Microbial Technology, Shandong University, Qingdao, 266237 People’s Republic of China; 2 CAS Key Lab of Biobased Materials, Qingdao Institute of Bioenergy and Bioprocess Technology, Chinese Academy of Sciences, Qingdao, 266101 People’s Republic of China

**Keywords:** Heme biosynthesis, Biosensor-based high-throughput screening, In vivo evolution, Protoporphyrin IX

## Abstract

**Background:**

Heme has attracted much attention because of its wide applications in medicine and food. The products of genes *hemBCDEFY* convert 5-aminolevulinic acid to protoporphyrin IX (PPIX; the immediate precursor of heme); protoporphyrin ferrochelatase (FECH) inserts Fe^2+^ into PPIX to generate heme. Biosynthesis of heme is limited by the need for optimized expression levels of multiple genes, complex regulatory mechanisms, and low enzymatic activity; these problems need to be overcome in metabolic engineering to improve heme synthesis.

**Results:**

We report a heme biosensor-guided screening strategy using the heme-responsive protein HrtR to regulate *tcR* expression in *Escherichia coli*, providing a quantifiable link between the intracellular heme concentration and cell survival in selective conditions (i.e., the presence of tetracycline). This system was used for rapid enrichment screening of heme-producing strains from a library with random ribosome binding site (RBS) variants and from a FECH mutant library. Through up to four rounds of iterative evolution, strains with optimal RBS intensities for the combination of *hemBCDEFY* were screened; we obtained a PPIX titer of 160.8 mg/L, the highest yield yet reported in shaken-flask fermentation. A high-activity FECH variant was obtained from the saturation mutagenesis library. Fed-batch fermentation of strain SH20C, harboring the optimized *hemBCDEFY* and the FECH mutant, produced 127.6 mg/L of heme.

**Conclusion:**

We sequentially improved the multigene biosynthesis pathway of PPIX and performed in vivo directed evolution of FECH, based on a heme biosensor, which demonstrated the effectiveness of the heme biosensor-based pathway optimization strategy and broadens our understanding of the mechanism of heme synthesis.

**Supplementary Information:**

The online version contains supplementary material available at 10.1186/s13068-023-02285-4.

## Introduction

Heme is a versatile and ubiquitous member of the tetrapyrrole family [1]. It plays important roles in gas sensing [2], signal transduction [3], transcriptional regulation [4], and oxygen transport [5]. Additionally, heme is a crucial cofactor of cytochromes in the electron transport chain, participating in aerobic and anaerobic respiration and photosynthesis [6]. Heme also acts as an essential cofactor for catalysis in various enzymes, such as cytochromes P_450_, catalase, and peroxidase [7, 8]. Reports in recent years have shown that heme can be an important preparation for meat seasoning [9]. As a US Food and Drug Administration-approved porphyria treatment drug, heme also has important applications in medicine. Therefore, there is a need for efficient production of heme [10, 11].

Heme is biosynthesized from the precursor 5-aminolevulinic acid (ALA) [12]. ALA can be condensed from succinyl-CoA and glycine, catalyzed by ALA synthase (ALAS) [13]. ALA can also be synthesized de novo from glucose as the sole carbon source [14] via the C5 pathway [15]. In the C5 pathway, glutamate is converted to ALA under the catalysis of glutamyl-tRNA synthetase (encoded by *gltX*), glutamyl-tRNA reductase (encoded by *hemA*), and glutamate-1-semialdehyde aminotransferase (encoded by *hemL*) [16]. Two molecules of ALA produce porphobilinogen (PBG) under the action of ALA dehydratase (encoded by *hemB*). Four molecules of PBG are polymerized to hydroxymethylcholane (HMB) by hydroxymethylcholane synthase (encoded by *hemC*). Uroporphyrinogen III synthase (encoded by *hemD*) catalyzes formation of uroporphyrinogen III (Uro III) from HMB. Uro III is converted to protoporphyrin IX (PPIX) under the catalysis of uroporphyrinogen decarboxylase (encoded by *hemE*), coproporphyrinogen III oxidase (encoded by *hemF*), and coproporphyrinogen III oxidase (encoded by *hemY*). Protoporphyrin ferrochelatase (FECH, encoded by *hemH*) then inserts Fe^2+^ into PPIX to generate heme (Fig. 3a) [17, 18].

A few reports have investigated the production of heme by microorganisms [1, 19]. Engineered *Escherichia coli* constitutively overexpressing ALAS and the downstream heme biosynthesis genes can produce 3.3 μM heme [20] using glycine and succinate as the carbon sources. The overexpression of heme pathway genes [e.g., *hemB*, *hemC*, *hemD*, *hemE*, and *coaA* (which encodes pantothenate kinase)] increased the heme concentration in *E. coli* [21]. Ko et al. overexpressed a noncanonical heme biosynthesis pathway in *Corynebacterium glutamicum*; heme production was enhanced by decreasing heme binding to cell membranes and upregulating pathway gene expression using systems metabolic engineering [22].

Although some advancements have been made in the synthesis of heme, significant challenges for heme production remain. For example, the regulatory mechanism of the heme biosynthesis pathway is complicated and not well understood. Simple overexpression of a gene may not achieve positive effects [1]. When the expression of *hemD* decreases, HMB will spontaneously convert to uroporphyrinogen I, thereby decreasing the synthesis of Uro III and thus heme. High levels of *hemD* and *hemF* expression result in increased ALA accumulation [17, 20, 23, 24]. These findings indicate that the expression level of each gene of the heme synthesis pathway needs to be fine-tuned. Multiple gene expression in the heme synthesis pathway was formerly optimized by sequentially changing plasmid copy numbers [25], but this is an a priori method that cannot produce an optimal design. FECH is considered to be a bottleneck in heme biosynthesis [1]. FECH derived from *Bacillus subtilis* was reported to have higher enzyme activity and achieved better heme titer than that from *E. coli* [25]. In vivo directed evolution of FECH is necessary to enhance the enzyme activity and heme synthesis in *E. coli*.

The challenges of systematic optimization of multigene pathways and enzyme directed evolution include needing to select or screen the optimum strain or enzyme from a randomly assembled library within a short time [26]. To meet this challenge, it is necessary to develop effective high-throughput screening tools [27–30]. Biosensors are amendable for use in high-throughput assays because they can provide a quantifiable link between genotype and phenotype by converting hard-to-quantify phenotypes to easily measurable parameters, such as fluorescence or growth [31, 32]. Here, we established a heme-responsive biosensor [33, 34]. We then used it for in vivo pathway optimization and directed evolution at the agar plate- and 96-well deep-well plate-scale for rapid screening of high-level heme producers from large libraries. We demonstrated the feasibility of the heme biosensor-based screening system in *E. coli* by sequentially improving the heme biosynthesis pathway. Heme production reached 127.6 mg/L in 5-L fed-batch fermentation.

## Results and discussion

### Establishing the heme biosensor-coupled screening system

The main challenge in establishing effective high-throughput screening tools is the rapid and accurate capture of high-performance strains from large libraries. Therefore, the ideal biosensor for our purposes would convert the accumulation of heme to a more easily measurable parameter, such as fluorescence or growth. We chose *tcR*, encoding the tetracycline efflux protein, as the output reporter gene. The previously developed heme biosensor HrtR was used as a detector. The DNA-binding site of HrtR, HrtO, was placed upstream of *tcR*, thereby coupling *tcR* expression intensity to heme concentration, generating the screening plasmid PHT.

**Fig. 1:**
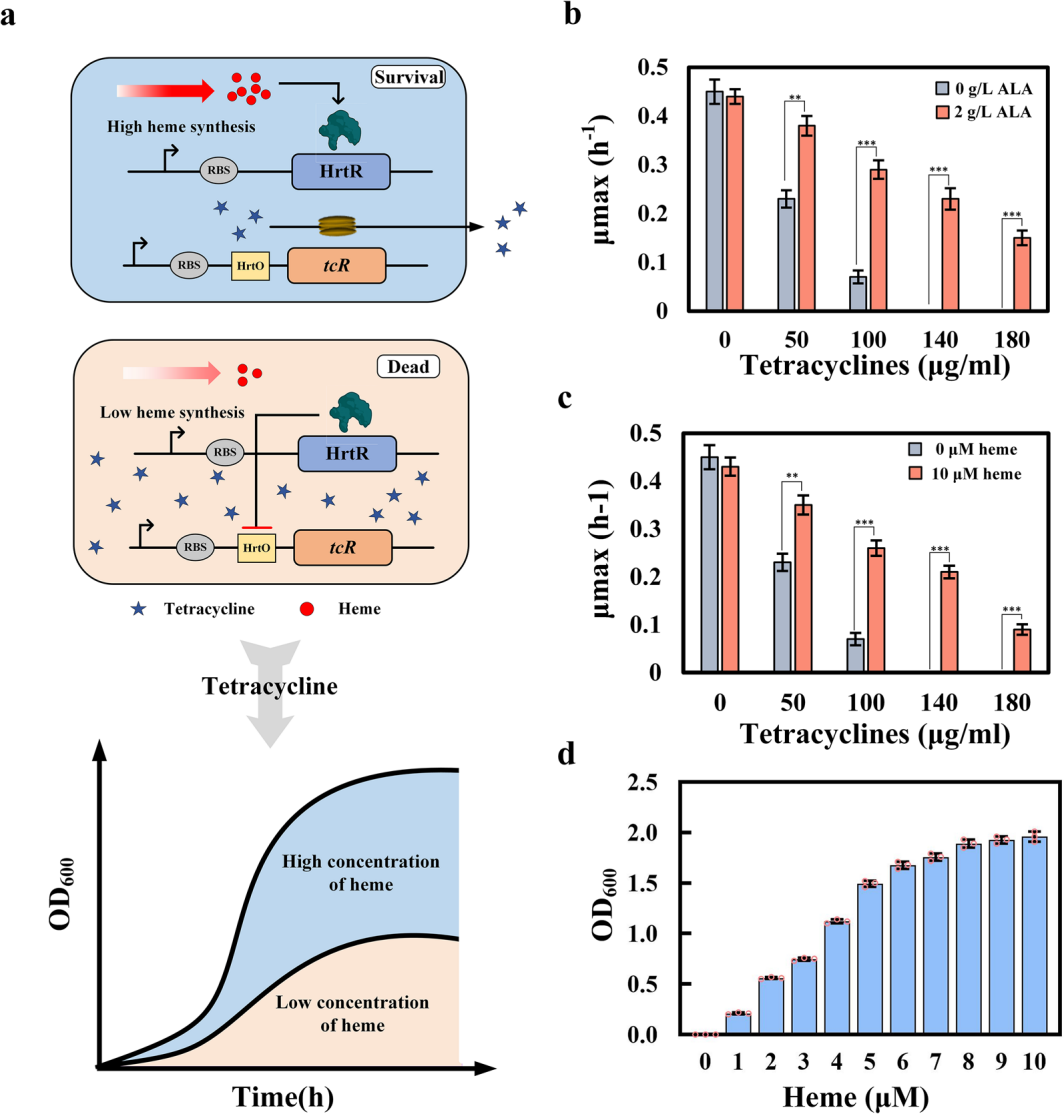
**a**: Schematic illustration of the heme biosensor-coupled screening system. When the heme concentration is high, HrtR relieves the inhibition of *tcR*, tcR effluxes tetracycline, and the cells grow well. When the heme content is low, HrtR inhibits *tcR* expression, and excessive tetracycline inhibits the growth of the cells; **b**: Specific growth rates of SPHT at different tetracycline concentrations on addition of 5-aminolevulinic acid (ALA); **c**: Specific growth rates of SPHT at different tetracycline concentrations on addition of heme; **d**: Growth differences of SPHT caused by heme addition in the presence of 140 μg/mL tetracycline. *0.01 < p < 0.05, **0.001 < p < 0.01, ***p < 0.001

As shown in Fig. 1a, the *tcR* expression controlled by heme biosensor HrtR couples the in vivo heme concentration to the ability of cells to resist tetracycline. When the in vivo concentration of heme is low, the activated HrtR inhibits the expression of *tcR*; thus, the cells cannot resist a high concentration of tetracycline. When the in vivo concentration of heme is high, heme binds to HrtR and it dissociates from promoter to turn on the expression of *tcR*, so that much tetracycline can be excreted outside the cell, keeping the cells alive. On the basis of this system, strains with high heme synthesis ability can be isolated based on their growth state in the presence of tetracycline.

Before high-throughput screening, the screening system pHT was evaluated in *E. coli* BL21 (DE3), generating strain SPHT. Since the addition of heme requires the overexpression of transporters, ALA was first selected to increase the concentration of intracellular heme. SPHT cells were cultured in medium supplemented with 0 or 2 g/L of ALA and 10–250 μg/mL tetracycline (Fig. 1b). In ALA-free medium, the specific growth rates of the strain were not affected when the tetracycline concentration was  < 20 μg/mL. When the tetracycline concentration was  > 20 μg/mL, the growth of the strain was restricted, and the strain stopped growing at 140 μg/mL tetracycline. The addition of ALA provided precursor for heme synthesis and thus enhanced the ability of the strain to resist tetracycline. At 140 μg/mL tetracycline, the specific growth rate of SPHT with 2 g/L of ALA was 0.21 h^−1^, while the control did not grow at this drug concentration. The OD_600_ of SPHT with 2 g/L of ALA reached 1.65, eightfold that of the control (Additional file 6: Figure S2a). When the tetracycline concentration was  > 140 μg/mL, the growth of strain SPHT with 2 g/L of ALA was significantly restricted and the specific growth rate was reduced (Fig. 1b, Additional file 6: Figure S2a).

To test the dose–response of this HrtR-based screening system toward heme, the heme transporter ChuA (derived from *E. coli* O157:H7 EDL933) was overexpressed in SPHT to enhance its heme uptake ability (Additional file 6: Figure S2c). The addition of 10 μM heme to the culture medium greatly enhanced the viability of this strain in the presence of tetracycline; the OD_600_ after culture under 140 μg/mL tetracycline for 12 h was 1.76, 10 times that of the strain with no heme addition (Additional file 6: Figure S2b). Furthermore, the addition of heme caused the greatest difference in the specific growth rate of SPHT at 140 μg/mL tetracycline (Fig. 1c). Then, different concentrations of heme (from 1 to 10 μM) were added to culture of SPHT in the presence of 140 μg/mL tetracycline, and it was found that the concentration of heme was positively correlated with the cell growth. When the addition of heme was  > 8 μM, the OD_600_ of the strain reached 2 (Fig. 1d). These results demonstrate that the heme biosensor-coupled high-throughput screening system was effective, and that 140 μg/mL tetracycline was most suitable concentration for growth screening because it caused maximum growth differentiation.

### Biosensor-driven optimization of multigene expression for balanced metabolic flux in PPIX biosynthesis

**Fig. 2:**
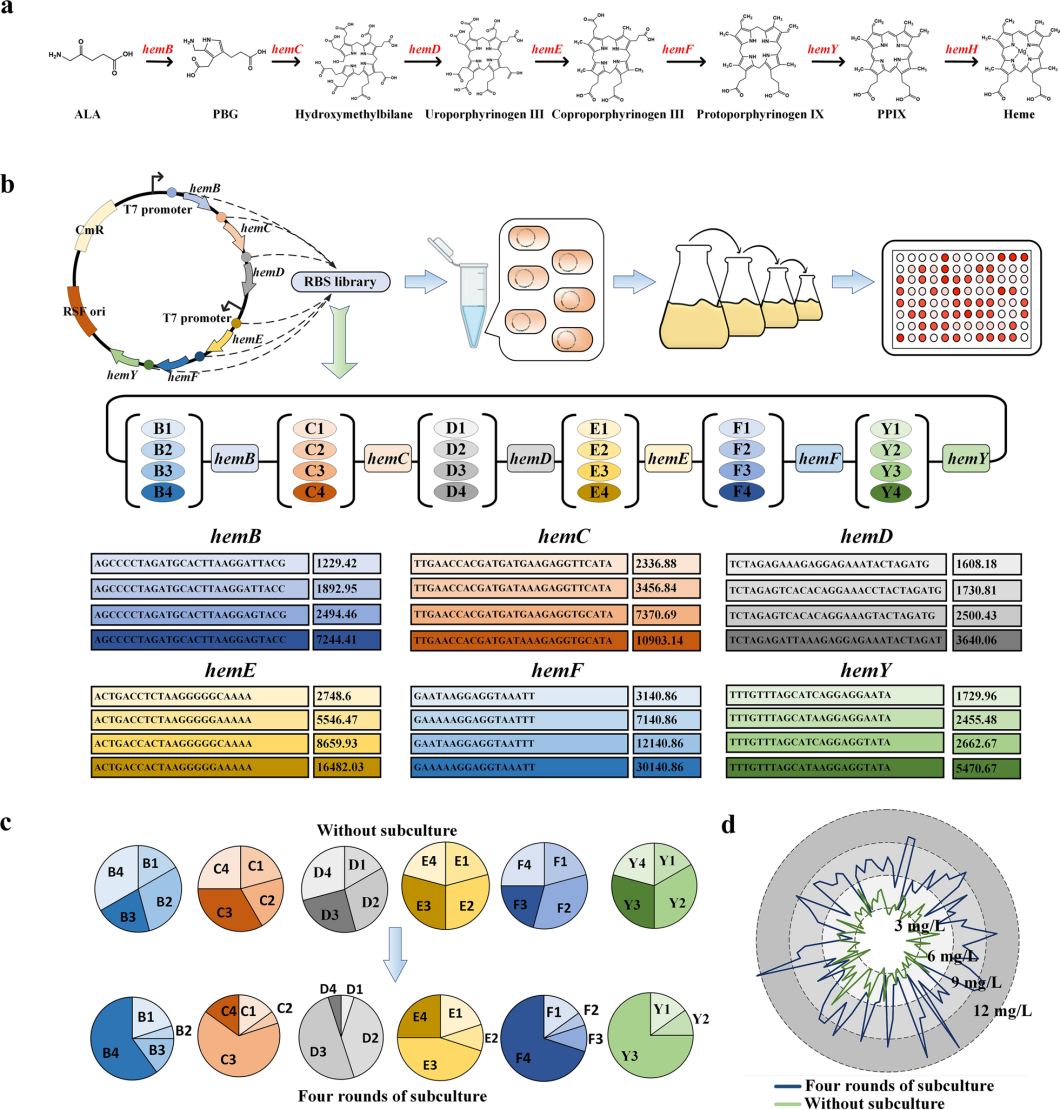
**a**: Schematic diagram of the heme synthesis pathway; **b**: Schematic diagram of the growth-coupled screening process and the designed ribosome binding site (RBS) library. Transcription of hemBCDEFY was initiated using two T7 promoters with four different intensities of RBS for each gene. B1–B4 represent the enhancement of strength, and the same for other genes. A darker color also represents an increase in intensity; **c**: RBS statistics for initial strains and strains following four rounds of subculture; **d**: PPIX production of initial strains and strains following four rounds of subculture in 96-well plates

An important principle of metabolic engineering is to balance the distribution of metabolic fluxes to obtain the desired phenotype [35]; this is also an effective means of circumventing the regulatory mechanisms in heme production. Because of the complexity of heme detection, traditional metabolic modification is time consuming and labor intensive. It was reported that due to the complex regulation of the heme biosynthesis pathway, the high level of expression of *hemD* and *hemY* was unfavorable for the accumulation of PPIX [23]. Natural FECH has low enzyme activity; changing transcription or translation efficiency on this basis has limited effect on improving heme synthesis efficiency. At the same time, in addition to being a direct precursor of heme, PPIX is also a high value drug precursor and a precursor in the synthesis of essential prosthetic tetraporphyrin groups Chl. The high level of accumulation of PPIX has other significance besides the production of heme. Therefore, we chose PPIX as the node for metabolic flux optimization. To optimize the expression intensities of genes involved in PPIX synthesis, genes *hemBCDEFY* were divided into two operons, and each gene was expressed using low, medium–low, medium–high, and high intensities of ribosome binding site (RBS), respectively. RBSs with different intensities were obtained using the library calculation function of RBS calculator (Fig. 2a, b). The total capacity of the library was 4^6^ = 4096.

The six genes with the designed different RBSs, *hemBCDEFY*, were cloned into pRSFduet-1-derived plasmids using Gibson assembly to construct the plasmid library. *hemA* and *hemL*, which enhance the synthesis of ALA, and *hemH*, which encodes FECH that converts the produced PPIX into heme, were overexpressed in pHT to form plasmid pHTALH. Then, the plasmid library was transformed into *E. coli* BL21 (DE3) harboring pHTALH. Then, we randomly selected 20 single colonies for sequencing from the unpassaged strains. The results showed that the library evenly included RBSs of each intensity, and the RBSs of different intensities were evenly distributed, which confirmed that the constructed library contained all the RBSs (Fig. 2c).

The strains containing the library plasmid and pHTALH were serially passaged under screening pressure to enrich for high-yielding strains. After four rounds of subculture, the plasmids were extracted. pHTALH contains a specific restriction enzyme site, *Eco*72I, which is absent from the library plasmid. Therefore, *Eco*72I can be used to remove pHTALH. After pHTALH was removed, the remaining plasmids were retransformed into *E. coli* BL21 (DE3). The obtained strains were streaked on LB agar plates, 96 single colonies were randomly selected for fermentation in 96-well plates, and the production of PPIX was determined. After this enrichment, most of the screened strains reached a PPIX yield of 9 mg/L (Fig. 2d, Additional file 7: Figure S3). The average PPIX accumulation of the fourth-generation strains was higher than that of the strains before screening (PPIX yield of 3–7 mg/L) (Fig. 2d). Simultaneously, on the basis of the 96-well plate fermentation results, 20 strains with high PPIX yields after fourth-generation enrichment were selected and the plasmids were sequenced. The RBS distributions of the six genes showed obvious biases (Fig. 2c). Among them, the RBSs of *hemB* were mainly (60% of the sequenced isolates) of the highest intensity B4. The RBSs of *hemF* were mainly (70%) of F4. These data indicate that high-intensity expression of *hemB* and *hemF* was beneficial for the conversion of ALA to PPIX. The RBSs of *hemC* were mainly of C3. The RBSs of *hemE* were mainly of E3 and E4. The RBSs of *hemY* were mainly of Y3; the highest intensity RBS (Y4) was not detected among the 20 strains. These findings indicate that medium-to-high level expression of *hemC*, *hemE*, and *hemY* facilitated the accumulation of PPIX. The RBSs of *hemD* were mainly of D2 and D3 (i.e., moderate intensity). Our results showed that the optimal RBS combination was successfully enriched by the heme biosensor-coupled growth iteration screening strategy.

**Fig. 3:**
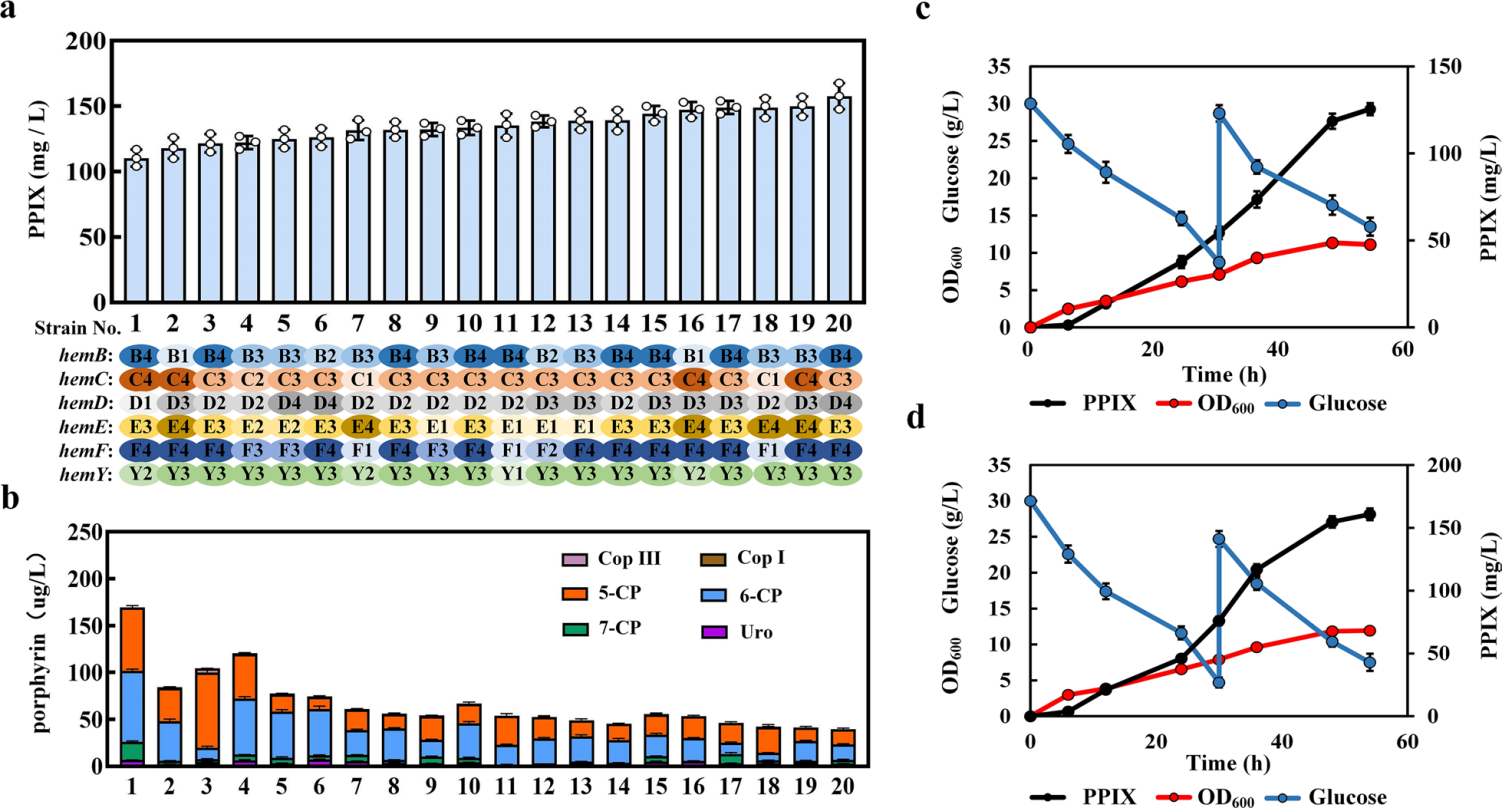
**a**: PPIX production and RBS distribution in the screened strains; **b**: porphyrin accumulation during PPIX production; **c**: PPIX production of strain No. 20 using glucose as the carbon source; **d**: PPIX production of strain No. 20 using glucose as the carbon source after optimization of fermentation conditions

The above 20 fourth-generation strains were fermented in shaken flasks with 4 g/L ALA added to MR-Fe20 medium. The accumulation of PPIX was determined after 54 h of fermentation. The PPIX yields of the 20 strains were all > 100 mg/L, and the highest yield, from strain No. 20, was 157.78 mg/L (Fig. 3a). Meanwhile, porphyrins also accumulated during the conversion of ALA to PPIX. The six porphyrin compounds found in the fermentation broth were uroporphyrin, heptaporphyrin, hexaporphyrin, pentaporphyrin, CopI, and CopIII. The accumulation of porphyrins decreased as the production of PPIX increased. To avoid the addition of the expensive precursor ALA for PPIX synthesis, pDAL [36] was transformed into strain No. 20; the final accumulation of PPIX in this engineered strain was 125.4 mg/L (Fig. 3c). Then, the fermentation conditions were optimized by changing the seed culture conditions and prolonging the seed cultivation time. After these optimizations, the bacterial growth became faster, the glucose consumption increased, and the strain accumulated 160.8 mg/L PPIX (Fig. 3d), which is the highest yield yet reported in shaken-flask fermentation. The results showed that metabolic flux balance had significant effect on PPIX production. Meanwhile, compared with modifying plasmid copy number or promoter strength, high-throughput screening is more efficient to obtain the ideal variant. We successfully constructed a heme biosensor-based in vivo pathway optimization method and used directed evolution to capture improved heme producers. This method further demonstrates the importance of flux balance in the heme synthesis pathway and provides new ideas for remodeling the heme metabolic pathway.

### In vivo directed evolution of FECH for improved heme biosynthesis

**Fig. 4:**
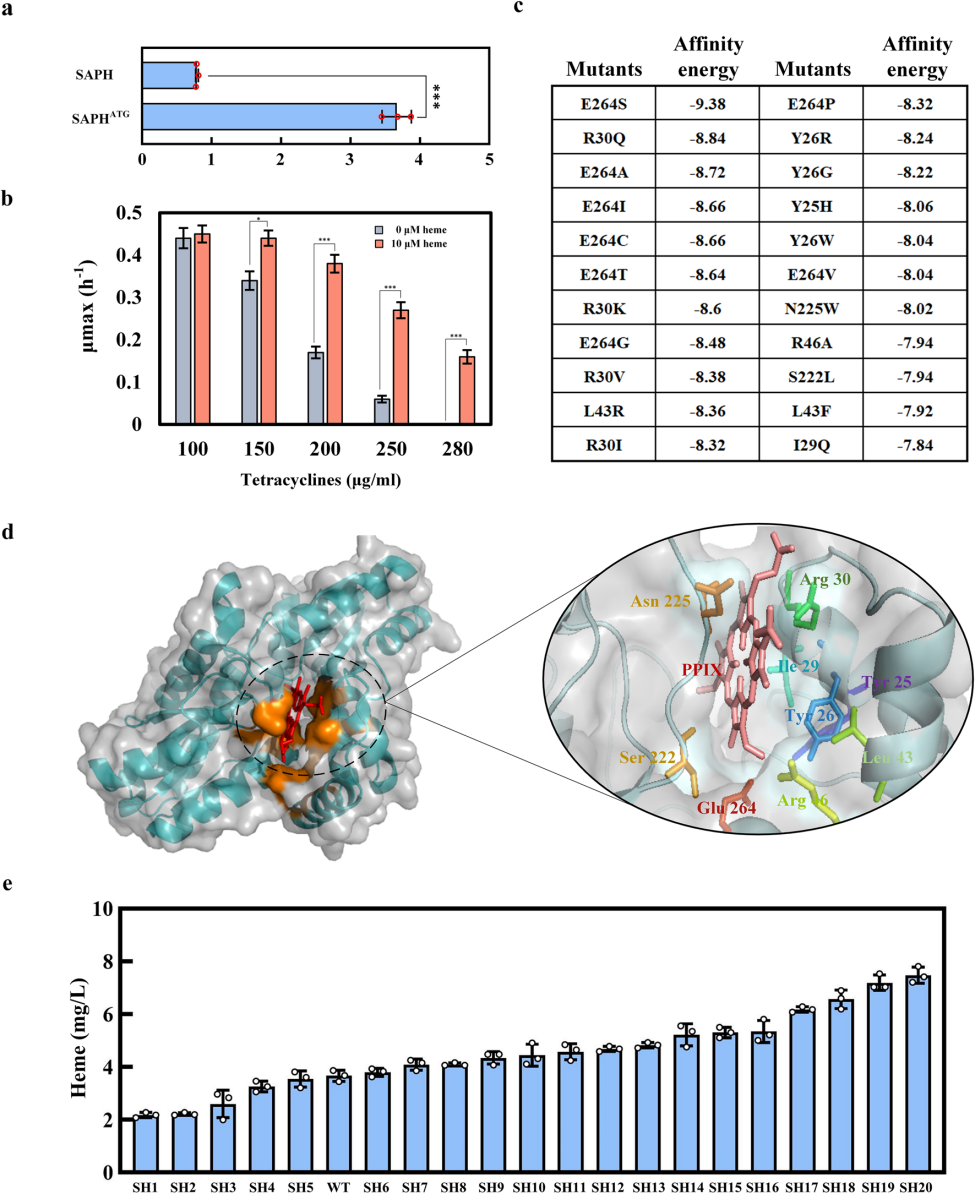
**a**: Heme yield after initiation codon optimization of *hemH*; **b**: Specific growth rates of SALHT at different tetracycline concentrations with heme addition **c**: Computer-aided design of mutants and affinity energies for FECH; **d**: Molecular docking model of PPIX and FECH from *Bacillus subtilis*; **e**: The heme yields of 20 strains were randomly selected from the pressure-screened FECH mutants. *0.01 < p < 0.05, **0.001 < p < 0.01, ***p < 0.001

FECH catalyzes Fe ion chelation in heme synthesis [1], while elaborate enzyme engineering of FECH has not been achieved. Thus, the most effective enzyme engineering strategy is to screen for ideal FECH variants in mutant libraries. To further facilitate the accumulation of heme, we overproduced FECH from *B. subtilis* in strain No. 20, forming strain SAPH. Strain SAPH produced 0.79 mg/L of heme after 54 h of shaken-flask fermentation (Fig. 4a). The initiation codon of *hemH* (encoding FECH) in strain No. 20 was GTG. We added a new start codon, ATG, before this GTG to obtained strain SAPH^ATG^. Strain SAPH^ATG^ produced a heme titer of 3.67 mg/L (Fig. 4a); subsequent engineering will be carried out on the basis of SAPH^ATG^.

The conversion rate of PPIX to heme was limited, and therefore, we attempted to use the biosensor-coupled screening strategy to evolve FECH for increased heme biosynthesis. *hemA*, *hemL*, and wild-type *hemH* after initiation codon optimization were overexpressed from plasmid pALH. pALH and the heme concentration screening plasmid pHT were co-transformed into strain No. 20 to obtain strain SALHT. To determine the appropriate tetracycline concentration for screening, strain SALHT was cultured in medium supplemented with 10 μM heme and 10–350 μg/mL tetracycline (Fig. 4b). In the absence of added heme, the growth of the strain was unaffected when the tetracycline concentration was  < 130 μg/mL; the growth gradually decreased as the tetracycline concentration was increased above 130 μg/mL, and it stopped at 280 μg/mL tetracycline. Adding 10 μM heme significantly improved the growth of the strain, and the OD_600_ value reached 1.45 at 280 μg/mL tetracycline (Additional file 8: Figure S4). The specific growth rate in the presence and absence of heme showed the greatest difference at 280 μg/mL tetracycline (Fig. 4b). Therefore, a tetracycline concentration of 280 μg/mL was selected for FECH screening.

**Fig. 5:**
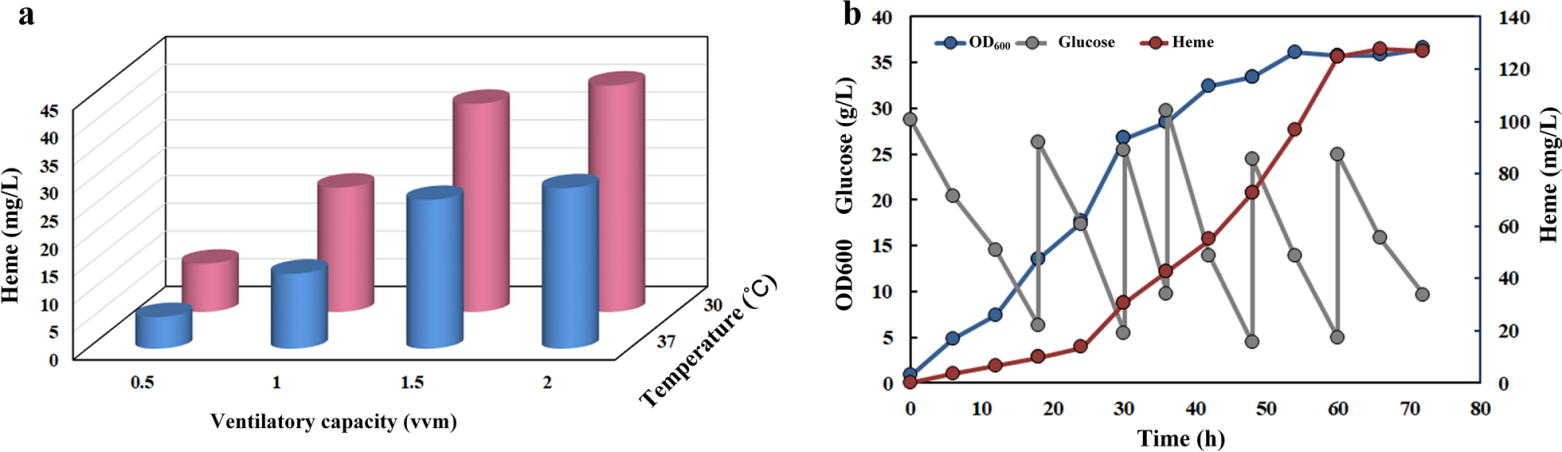
**a**: Parallel fermentations to optimize heme fermentation conditions; **a** 1-L fermenter was used with medium volume 600 mL and pH stabilized at 7.0; **b**: Heme production in a 5-L fermenter. The temperature was 30 °C, the ventilation volume was 2 vvm, and the pH was stabilized at 7.0

The mutants with improved PPIX affinity of FECH were screened by modification of enzyme activity based on molecular dynamics simulation and molecular docking (data not shown). Nine potentially desirable mutations of *B. subtilis* FECH were identified: in residues Y25, Y26, I29, R30, L43, R46, S222, N225, and E264 (Fig. 4c, d). A mutant library of FECH was obtained by combining these mutations. *hemH* was mutated used mutation primer (Additional file 3: Table S3) in pALH, and the obtained mutant library and pHT were co-transformed into strain No. 20. The resulting mutant strains were sub-screened under screening pressure (280 μg/mL tetracycline) to enrich for high-heme-producing strains. Twenty strains (named strains SH1–SH20, respectively) were randomly selected after five rounds of subculture for shaken-flask fermentation (Additional file 4: Table S4). Among them, strains SH6–SH20 had higher heme production than the wild type; the yield of strain SH20 reached 7.48 mg/L, which was 2.04-fold that of the wild type. Through sequencing, it was found that FECH in SH20 contained seven mutations compared with the wild type: Y26V, I29T, R30E, L43R, R46A, S222R, and E264L. According to the molecular docking model, the mutated sites are located in the PPIX-binding cavity, indicating that the modification of key sites for substrate binding by FECH is an effective way to improve the catalytic activity of the enzyme. Because docked structures of FECH and PPIX have not been resolved, we predicted the key sites for substrate binding by FECH by molecular docking, used computer-aided design of mutations, and constructed a mutant library accordingly, from which we successfully screened FECH variants with higher activity. Compared with holoenzyme mutation, constructing a mutant library on the basis of computer-aided design avoids the generation of a large number of undesired variants and improves the success rate of screening for ideal variants.*ccmABC*, encoding a cytoplasmic to periplasmic heme transport protein, was overexpressed in strain SH20 to enhance heme export, thereby reducing the toxicity caused by excessive accumulation of heme in the cytoplasm, forming strain SH20C. Strain SH20C accumulated a heme titer of 8.2 mg/L in shaken flasks. Then, strain SH20C was cultivated in a 1-L fermenter for optimization of fermentation conditions (temperature and dissolved oxygen concentration). The results showed that 30 °C was the most suitable temperature for heme production, giving a higher yield than was obtained at 37 °C at the same ventilation volume (Fig. 5a). An increase in ventilation volume was also beneficial for heme production, and when the ventilation was 2 vvm, the heme production reached 40.8 mg/L. SH20C was further cultured in a 5-L fermenter at 30 °C and 2 vvm. On adding Fe^2+^ and sodium glutamate, the heme yield reached 127.6 mg/L (Fig. 5b).

## Conclusions

In this study, we successfully constructed a heme biosensor-based in vivo pathway optimization method and used directed evolution to capture improved heme producers. Using this approach, we increased the yield of PPIX from ALA to 160.8 mg/L, which is the highest yield yet reported in shaken-flask fermentation. This method further demonstrates the importance of flux balance in the heme synthesis pathway and provides new ideas for remodeling the heme metabolic pathway. In addition, we predicted the key sites for substrate binding by FECH by molecular docking, used computer-aided design of mutations, and constructed a mutant library accordingly, from which we successfully screened FECH variants with higher activity. Compared with holoenzyme mutation, constructing a mutant library on the basis of computer-aided design avoids the generation of a large number of undesired variants and improves the success rate of screening for ideal variants. After multiple rounds of screening, and improvement of fermentation conditions, we increased the yield of heme to 127.6 mg/L. This work proves that biosensor-driven metabolic flux optimization and enzyme evolution engineering are effective strategies to increase heme yield. But this yield is still a long way from the target of commercialization. Supplementing the supply of precursors, further optimizing the enzyme activity of FECH and optimizing the fermentation conditions may be the focus of future work to increase the yield of heme. We enriched the application scope of heme biosensors and expanded the application of synthetic biology strategies in engineering heme metabolism.

## Materials and methods

### General procedures

The strains used in this study are summarized in Additional file 1: Table S1. Molecular cloning and manipulation of plasmids were performed using *E. coli* DH5α. The plasmids and oligonucleotides used in this work are listed in Additional file 2: Tables S2 and Additional file 3: Table S3. For PPIX and heme fermentation, *E. coli* strain BL21 and MR-Fe20 medium were used. MR-Fe20 medium contained 6.67 g/L KH_2_PO_4_, 4 g/L (NH4)_2_HPO_4_, 0.8 g/L MgSO_4_∙7H_2_O, 0.8 g/L citric acid, 20 mg/L FeSO_4_∙7H_2_O, and 5 mL of trace metal solution A per liter. Trace metal solution A contained 0.5 mol of HCl, 2 g/L CaCl_2_, 2.2 g/L ZnSO_4_∙7H_2_O, 0.5 g/L MnSO_4_∙4H_2_O, 1 g/L CuSO_4_∙5H_2_O, 0.1 g/L (NH_4_)_6_Mo_7_O_24_∙4H_2_O, 0.02 g/L Na_2_B_4_O_7_∙10H_2_O, and 10 g/L FeSO_4_∙7H_2_O [25] (Additional file 4: Table S4).

### Plasmid construction

Primers Cdfduet-F/R were used to amplify the replicon fragment from plasmid pCDFduet-1. The *tcR* fragment was amplified from pCDFduet-1 using primers TcR-F/R. The *hrtR* fragment was amplified from plasmid pSB using primers HrtR-F/R. The resulting fragments were assembled using Gibson assembly [37] to generate plasmid pHT. Using *B. subtilis* genomic DNA as the template and primers HemH-F/R, the *hemH* fragment was amplified. The *hemA-L* fragment was amplified from plasmid pDAL using primers hemA-F and hemL-R. The *hemH* and *hemA-L* fragments were assembled into plasmid pHT to obtain plasmid pHTALH.

### Library construction

Primers hemB-F/R, hemC-F/R, hemD-F/R, hemE-F/R, and hemF-F/R were used to amplify *hemB*, *hemC*, *hemD*, *hemE*, and *hemF* fragments, respectively, from *E. coli* genomic DNA. Among them, hemB-F, hemC-F, hemE-F, and hemY-F were degenerate primers. A total of four hemD-F primers, named hemD-F1–4, were mixed together to give primer “hemD-F.” The *hemY* fragment was amplified from *B. subtilis* genomic DNA using primers hemY-F/R. Using primers T7-hemB-F and hemD-R and the *hemB*, *hemC*, and *hemD* fragments, overlap PCR was performed to obtain a “BCD” fragment. Using primers T7-hemE-F and hemY-R, and the *hemE*, *hemF*, and *hemY* fragments, overlap PCR was performed to obtain an “EFY” fragment. Primers rsf-F/R were used to amplify a vector fragment from plasmid rsfduet-1. The obtained vector fragment, “BCD” fragment, and “EFY” fragment were assembled by Gibson assembly to construct library plasmid pBY.

Primers puc-F and puc-R were used to obtain the plasmid backbone fragment of pUC19. Primers hemA-F and hemL-R were used to amplify the *hemA-L* fragment from plasmid pDAL. Primers hemH-F and hemH-R were used to amplify *hemH* from *B. subtilis* genomic DNA. The resulting fragments were assembled using Gibson assembly to obtain plasmid pALH. We mutated *hemH* on the basis of pALH, cloned *hemH* into three fragments using three pairs of primers containing degenerate bases (mutat-F1/R1, mutat-F2/R2, and mutat-F3/R3), and then used overlap PCR to regenerate a *hemH* fragment containing random mutations. The wild-type *hemH* on pALH was replaced with the obtained fragment to obtain a library plasmid.

### Selection methods

In the screening of metabolic fluxes and FECH mutants, the library plasmid was transferred into strains containing the screening system. Antibiotics and corresponding tetracycline concentrations were added for passage, cultivated 12 h for one generation.

### Analytical methods

PPIX standards purchased from Sigma-Aldrich (St. Louis, MO, USA). PPIX was dissolved in DMSO to obtain 10, 20, 30, 40, 50, 60, 70, 80, 90, and 100 μg/L solutions, respectively. PPIX solutions of different concentrations (200 μL) were pipetted into a 96-well plate. A microplate reader was used to detect the fluorescence intensity of the solutions (excitation at 410 nm, emission at 633 nm). A standard curve was drawn (Additional file 5: Figure S1). Fermentation broth was centrifuged to obtain the supernatant. After appropriate dilution by DMSO, the fluorescence intensity (excitation at 410 nm, emission at 633 nm) was detected in 96-well plates, and the PPIX concentration was determined from the standard curve [38].

Heme concentration was determined using a high-performance liquid chromatography (HPLC) system with a Discovery HS C18 column (250 × 4.6 mm, 5 μm; Supelco Inc., USA). Filtered samples were separated using a linear gradient of 20%–95% solvent A in B at 40 °C. Solvent A was a 10:90 (v/v) HPLC-grade methanol:acetonitrile mixture, and solvent B was 0.5% (v/v) trifluoroacetic acid in HPLC-grade water. The flow rate was 1 mL/min for 40 min, and the absorbance was measured at 400 nm [39]. When the heme concentration was  < 2 mg/L, a Heme Colorimetric Assay Kit (BioVision, USA) was used for determination.

ALA and PBG concentrations were analyzed using modified Ehrlich’s reagent [36]. Porphyrin was determined by HPLC. The fermentation broth was extracted twice with 5% HCl and incubated at 37 °C for 30 min. After centrifugation, the supernatant was passed through a Bischoff Prontosil 120-5-C18 ace EPS chromatography column (125 mm × 4 mm; 5 μm) at a flow rate of 0.75 mL/min. Porphyrin was detected at 30 °C using a fluorescence detector (excitation 400 nm, emission 620 nm). Mobile phase A was 1 M ammonium acetate, pH 5.16, with 10 mL triethylamine and 100 mL acetonitrile in 1-L chromatographic-grade water; mobile phase B was methanol:acetonitrile (9:1, v:v). During the first 15 min, the proportion of mobile phase A was a linear decreased from 62 to 5%; the mobile phase A ratio was then maintained at 62% for 10 min.

### Fed-batch fermentation

Strains were cultured overnight in Luria–Bertani medium for 18 h, transferred to MR-FE20 medium at 4% inoculum for 18 h, and then inoculated into parallel fermenters at 10% inoculum. The pH was adjusted with 1 M sulfuric acid and ammonia water. Samples were taken every 6 h to determine OD_600_ values, and glucose and product concentrations. If the glucose concentration was  < 10 g/L, the fermenter was supplemented with 20 g/L glucose.

MR-FE20 medium (3 L) was placed in a 5-L fermenter. Strains were cultured overnight in LB medium for 18 h, transferred to MR-FE20 medium at 4% inoculum for 18 h, and then inoculated into the fermenter at 10% inoculum. The temperature was 30 °C and the ventilation volume 2 vvm. Sampling time, pH regulation, and the feeding method were consistent with the experiments in parallel fermenters.

## Supplementary Information

**Additional file 1: Table S1.** Bacterial strains used in this study.

**Additional file 2: Table S2.** Plasmids used in this study.

**Additional file 3: Table S3.** Primers used in this study.

**Additional file 4: Table S4.** SH1–SH20 mutation.

**Additional file 5: Figure S1.** Protoporphyrin IX (PPIX) standard curve.

**Additional file 6: Figure S2.** a: OD_600_ of SPHT at different tetracycline concentrations on addition of 5-aminolevulinic acid (ALA); b: OD_600_ of SPHT at different tetracycline concentrations on addition of heme; c: The heme transporter ChuA (derived from E. coli O157:H7 EDL933) was overexpressed in SPHT.

**Additional file 7: Figure S3.** a: PPIX production of initial strains of subculture in 96-well plates; b: PPIX production of strains following four rounds of subculture in 96-well plates.

**Additional file 8: Figure S4.** OD_600_ of SALHT at different tetracycline concentrations with heme addition.

## Data Availability

All data generated or analyzed during this study are included in this published article and its supplementary materials.
