# Supplementary material for: Heme biosensor-guided in vivo pathway optimization and directed evolution for efficient biosynthesis of heme
Source: Biotechnol Biofuels Bioprod. 2023 Mar 1;16:33. doi: 10.1186/s13068-023-02285-4 (PMC9979517; doi:10.1186/s13068-023-02285-4)
Supplement: Supplementary file 1 — Additional file 1: Table S1. Bacterial strains used in this study. [file 13068_2023_2285_MOESM1_ESM.docx]

**Table S1: Bacterial strains used in this study.**

| **Strains** | **Relevant properties** | **Source** |
| --- | --- | --- |
| **DH5α** |  | **lab stock** |
| **BL21(DE3)** |  | **lab stock** |
| ***B. subtilis*** |  | **lab stock** |
| **SPHT** | **BL21(DE3) harboring pHT** | **this study** |
| **SBY** | **BL21(DE3) harboring library plasmid pBY** | **this study** |
| **Strain No.1-20** | **20 randomly selected strains of SBY after screening** | **this study** |
| **S20AL** | **Strain No.20 harboring pDAL** | **this study** |
| **SAPH** | **Strain No.20 harboring pALH^bsu^** | **this study** |
| **SAPH^ATG^** | **Strain No.20 harboring pALH^atg^** | **this study** |
| **SALHT** | **Strain No.20 harboring pALH and pHT** | **this study** |
| **SHBY** | **Strain No.20 harboring pHT and pALH^library^** | **this study** |
| **SH1-SH20** | **20 randomly selected strains of SHBY after screening** | **this study** |
| **SH20C** | **Strain No.20 harboring pALH-20C** | **this study** |
