# Supplementary material for: Heme biosensor-guided in vivo pathway optimization and directed evolution for efficient biosynthesis of heme
Source: Biotechnol Biofuels Bioprod. 2023 Mar 1;16:33. doi: 10.1186/s13068-023-02285-4 (PMC9979517; doi:10.1186/s13068-023-02285-4)
Supplement: Supplementary file 2 — Additional file 2: Table S2. Plasmids used in this study. [file 13068_2023_2285_MOESM2_ESM.docx]

**Table S2: Plasmids used in this study.**

| **Plasmids** | **Charecteristics** | **Source** |
| --- | --- | --- |
| **prsf-duet** |  | **lab stock** |
| **pUC19** |  | **lab stock** |
| **pCDF-duet** |  | **lab stock** |
| **pDAL** |  | **lab stock** |
| **pHT** | **Pcdf-duet harboring HrtR and tcR** | **this study** |
| **pBY** | **ORIrsf -CM- hemB-hemY RBS mutant library** | **this study** |
| **pHTALH** | **PHT harboring *hemA*, *hemL* and *hemH*** | **this study** |
| **pALH^bsu^** | **pDAL harboring *hemH^bsu^*** | **this study** |
| **pALH^atg^** | **The start codon of *hemH* in pALH^bsu^ was optimized.** | **this study** |
| **pALH^library^** | **The *hemH* mutant library constructed on the basis of pALH^atg^** | **this study** |
| **pALH-20** | **The screened plasmid No. 20 from pALH^library^** | **this study** |
| **pALH-20C** | **PALH-20 harboring CCMABC** | **this study** |
