# Supplementary material for: Heme biosensor-guided in vivo pathway optimization and directed evolution for efficient biosynthesis of heme
Source: Biotechnol Biofuels Bioprod. 2023 Mar 1;16:33. doi: 10.1186/s13068-023-02285-4 (PMC9979517; doi:10.1186/s13068-023-02285-4)
Supplement: Supplementary file 3 — Additional file 3: Table S3. Primers used in this study. [file 13068_2023_2285_MOESM3_ESM.docx]

**Table S3: Primers used in this study.**

| **Primers** | **Sequence** |
| --- | --- |
| **cdfduet-f** | **gaccgggtctccgcaagtggcacttttcggggctatttaacgaccctgccc** |
| **cdfduet-r** | **acagtcgtattaaaaaaattaggacgttaaccttcaacccagtcagctcc** |
| **hrtr-f** | **gtcaagcatcggtcgagatcccggtgcctaatgagataggcaccccaggc** |
| **hrtr-r** | **tctagtactttcctgtgtgactctagatcttcatgacactgtgtcatca** |
| **tcr-f** | **atgaagatctagagtcacacaggaaagtactagatgatgaatagttcgacaaagatcgc** |
| **tcr-r** | **ccgaaaagtgccacttgcggagacccggtcttaagcacttgtctcctgtttactcc** |
| **hema-f** | **gtcaagcatcggtcgagatcccggtgcctaatgagataggcaccccaggc** |
| **heml-r** | **agtcacgacgttgtaaaacgac** |
| **hemb-f** | **aacaattccagcccctagatgcacttaaggaktacsatgacagacttaatccaacgccc** |
| **hemb-r** | **atcatcgtggttcaattaacgcagaatcttcttctcagcc** |
| **hemc-f** | **ggctgagaagaagattctgcgttaattgaaccacgatgatraagaggtkcataatgttagacaatgttttaagaattgc** |
| **hemc-r** | **ctagatcatgccggggcgtctccgt** |
| **hemd-f1** | **tgaagtctataacggagacgccccggcatgatctagagaaagaggagaaatactagatgatgagtatccttgtcacccg** |
| **hemd-f2** | **agtctataacggagacgccccggcatgatctagagattaaagaggagaaatactagatgatgagtatccttgtcacccg** |
| **hemd-f3** | **gaagtctataacggagacgccccggcatgatctagagtcacacaggaaagtactagatgatgagtatccttgtcacccg** |
| **hemd-f4** | **aagtctataacggagacgccccggcatgatctagagtcacacaggaaacctactagatgatgagtatccttgtcacccg** |
| **hemd-r** | **tacgggagctcgccttattgtaatgcccgtaaaagcgcatcg** |
| **heme-f1** | **gagcggataacaattccactgaccwctaagggggmaaaaatgaccgaacttaaaaacga** |
| **heme-f2** | **acgggcattacaataaggcgagctcccgtaatacgactcactataggggaattgtgagcggataacaattccactgacc** |
| **heme-r** | **ttagcggtgatactgttcagaca** |
| **hemf-f** | **gtggaggcagtgcatcgactgtctgaacagtatcaccgctaagaawaaggaggtaawttatgaaacccgacgcacacca** |
| **hemf-r** | **ttacacccaatccctgacct** |
| **hemy-f** | **aagtgagtttattaaggtcagggattgggtgtaatttgtttagcatmaggaggwataatgagtgacggcaaaaaacatg** |
| **hemy-r** | **cctaggttatagtttagcggccgcattcttatttagctgaataaataggtaagcgcgtc** |
| **cm-f** | **ataagaatgcggccgctaaactataacctaggctgctgccaccg** |
| **cm-r** | **acggctttgccgcggccctc** |
| **rsf-f** | **agggaagtgagagggccgcggcaaagccgtggcatgcagcgctcttccgc** |
| **rsf-r** | **caacttatatcgtatggggctgacttcaggtgggtgctacatttgaagagataaattgc** |
| **hemh-one** | **aagcatttcaggttcaggctttctgcctctthbtkwatgtgtcmvgtracgttcaatatcttcttccttataaggc** |
| **hemh-two** | **agaggcagaaagcctgaacctgaaatgcttcaagatykcaaagacssctacgaagcgattggcggcatt** |
| **hemh-three-2** | **ggcgtttccgaatatgctgtcggctggcaatyggaagggtggacgcctgatccttggctcgg** |
| **hemh-three** | **ggcgtttccgaatatgctgtcggctggcaatyggaagggaacacgcctgatccttggctcgg** |
| **hemh-four** | **ttgccagccgacagcatatt** |
| **hemh-six** | **gtgctttatgataatgattatgaatgcaaag** |
| **hemh-five** | **tttgcattcataatcattatcataaagcacsnntaagtgatccgcgacaaaccc** |
