# Supplementary material for: Heme biosensor-guided in vivo pathway optimization and directed evolution for efficient biosynthesis of heme
Source: Biotechnol Biofuels Bioprod. 2023 Mar 1;16:33. doi: 10.1186/s13068-023-02285-4 (PMC9979517; doi:10.1186/s13068-023-02285-4)
Supplement: Supplementary file 4 — Additional file 4: Table S4. SH1–SH20 mutation. [file 13068_2023_2285_MOESM4_ESM.docx]

**Table S4: SH1-SH20 mutation**

| Strain | Mutation | | | | | | | | |
| --- | --- | --- | --- | --- | --- | --- | --- | --- | --- |
| SH1 | Y25H | Y26L | I29S | R30Q | L43P | R46R | S222W | N225W | E264G |
| SH2 |  | Y26V | I29S | R30Q | L43C | R46P | S222R | N225W | E264P |
| SH3 |  | Y26V | I29S | R30I | L43C | R46P | S222R | N225W | E264R |
| SH4 | Y25H | Y26W |  | R30K | L43R |  | S222R | N225W | E264L |
| SH5 | Y25H | Y26W |  | R30K | L43R |  | S222R | N225W | E264L |
| SH6 |  |  | I29K | R30K | L43C | R46P | S222R | N225W | E264L |
| SH7 |  | Y26L | I29S | R30K | L43F |  | S222W | N225W | E264M |
| SH8 | Y25H | Y26L | I29T | R30K | L43C | R46P | S222R |  | E264L |
| SH9 |  | Y26V | I29S | R30K | L43C | R46P | S222R | N225W | E264L |
| SH10 |  | Y26R | I29T | R30I | L43C | R46P | S222R | N225W | E264R |
| SH11 |  | Y26V |  | R30K | L43C | R46P | S222R | N225W | E264L |
| SH12 | Y25H | Y26L | I29T | R30I | L43R |  | S222W | N225W | E264G |
| SH13 |  | Y26L | I29S | R30K | L43P | R46A | S222R | N225W | E264R |
| SH14 |  | Y26V | I29S | R30I | L43C | R46P | S222R | N225W | E264L |
| SH15 |  | Y26R | I29T | R30K | L43C | R46P | S222R | N225W | E264M |
| SH16 |  | Y26L | I29S | R30I | L43F | R46C | S222W | N225W | E264R |
| SH17 | Y25H | Y26L | I29S | R30K | L43R | R46P | S222W |  |  |
| SH18 | Y25H | Y26R | I29T | R30I | L43R |  | S222R |  |  |
| SH19 |  | Y26R | I29T | R30K | L43R | R46P | S222R | N225W | E264L |
| SH20 |  | Y26V | I29T | R30E | L43R | R46A | S222R |  | E264L |
