# Supplementary figures and images for: Heme biosensor-guided in vivo pathway optimization and directed evolution for efficient biosynthesis of heme
Source: Biotechnol Biofuels Bioprod. 2023 Mar 1;16:33. doi: 10.1186/s13068-023-02285-4 (PMC9979517; doi:10.1186/s13068-023-02285-4)

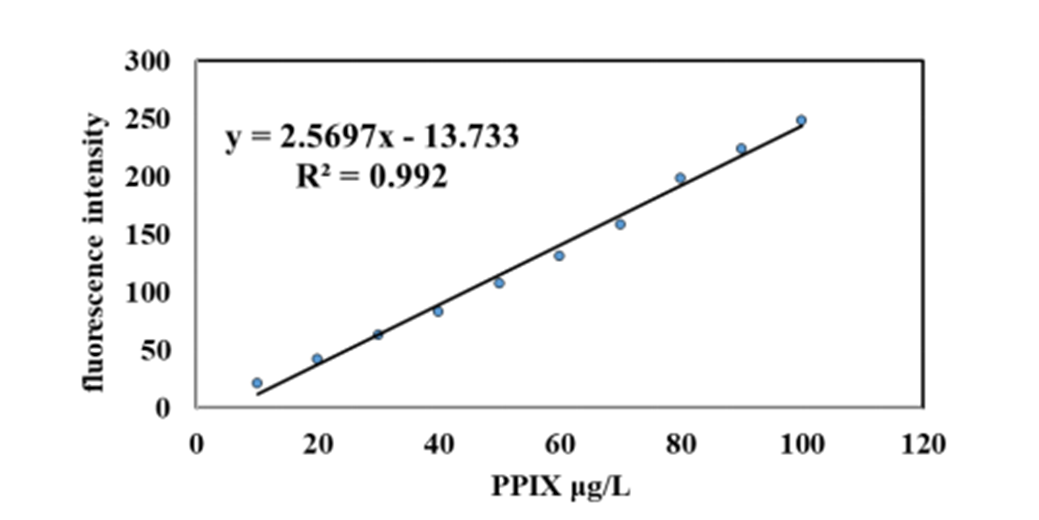

Supplement: Supplementary file 5 — Additional file 5: Figure S1. Protoporphyrin IX (PPIX) standard curve. [file 13068_2023_2285_MOESM5_ESM.tif]

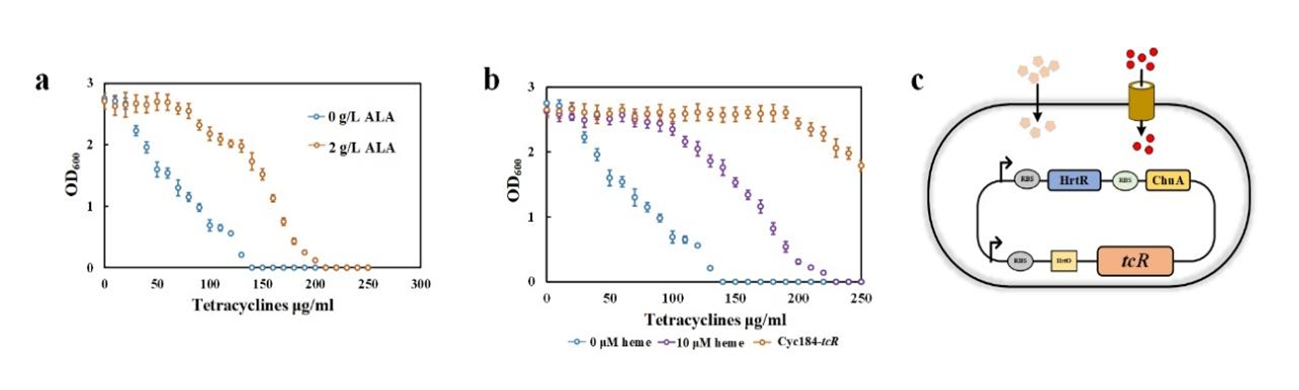

Supplement: Supplementary file 6 — Additional file 6: Figure S2. a: OD600 of SPHT at different tetracycline concentrations on addition of 5-aminolevulinic acid (ALA); b: OD600 of SPHT at different tetracycline concentrations on addition of heme; c: The heme transporter ChuA (derived from E. coli O157:H7 EDL933) was overexpressed in SPHT. [file 13068_2023_2285_MOESM6_ESM.tif]

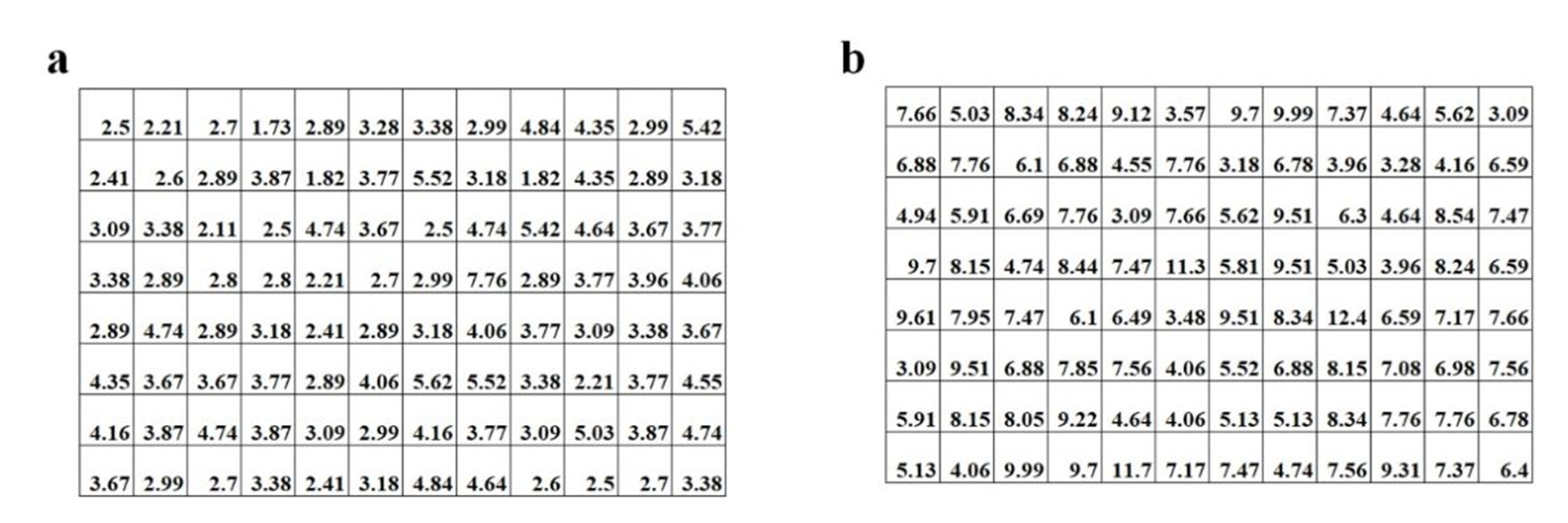

Supplement: Supplementary file 7 — Additional file 7: Figure S3. a: PPIX production of initial strains of subculture in 96-well plates; b: PPIX production of strains following four rounds of subculture in 96-well plates. [file 13068_2023_2285_MOESM7_ESM.tif]

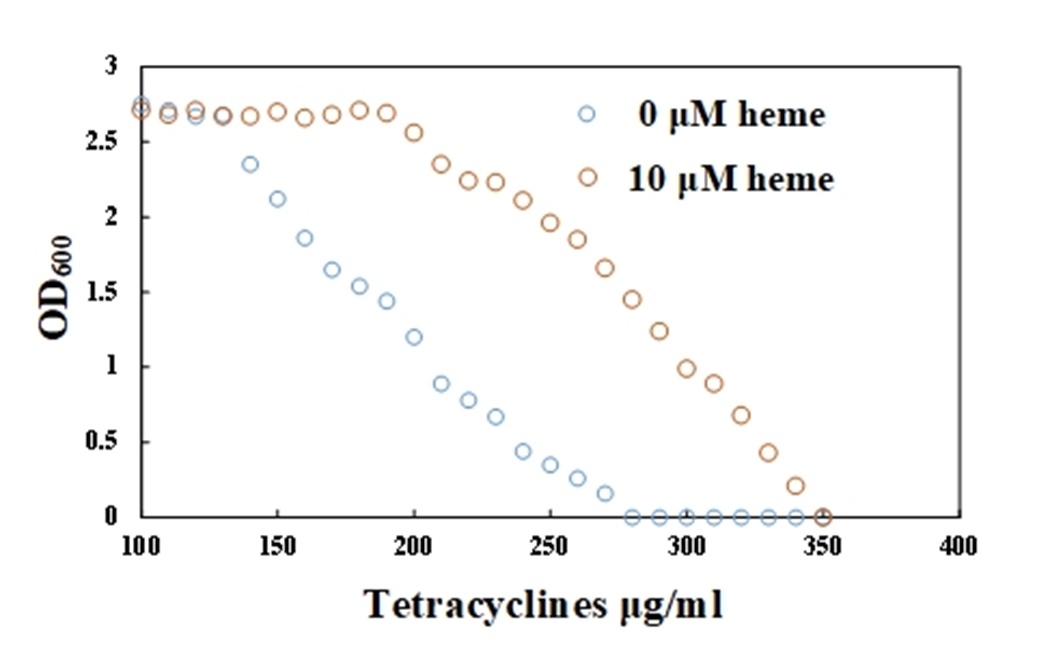

Supplement: Supplementary file 8 — Additional file 8: Figure S4. OD600 of SALHT at different tetracycline concentrations with heme addition. [file 13068_2023_2285_MOESM8_ESM.tif]
